# Oxygen vacancies of the TiO_2_ nano-based composite photocatalysts in visible light responsive photocatalysis

**DOI:** 10.1039/c8ra05117h

**Published:** 2018-10-04

**Authors:** Buanya Beryl Adormaa, Williams Kweku Darkwah, Yanhui Ao

**Affiliations:** Key Laboratory of Integrated Regulation and Resource Development on Shallow Lakes, Ministry of Education, Environmental Engineering Department, College of Environment, Hohai University Nanjing China andyao@hhu.edu.cn wkdarkwah@hhu.edu.cn williams.darkwah@stu.ucc.edu.gh +8613770610843

## Abstract

The TiO_2_ nano-based composite photocatalyst is best known for application in solving the recent issues related to energy and environmental purification. Due to the low cost, nontoxicity, chemical stability and high efficiency of TiO_2_, it is unquestionably one of the most considered materials in environmental treatment. In this systematic review, we reveal the outstanding potential of oxygen vacancy in photocatalysis, and discuss the contemporary advancement in the photocatalytic activities, productivity, preparation methods and oxygen vacancy of the TiO_2_ nano-based composite photocatalyst for environmental treatment and energy as well as wastewater treatment. This exposé is anticipated to enlighten researchers and engineers on the specific management and assessment of the environment, which warrants prospective research into developing appropriate mechanisms for energy, wastewater treatment and environmental purification.

## Abstract 2

The TiO_2_ nano-based composite photocatalyst is best known for application in solving the recent issues related to energy and environmental purification.

## Graphical abstract

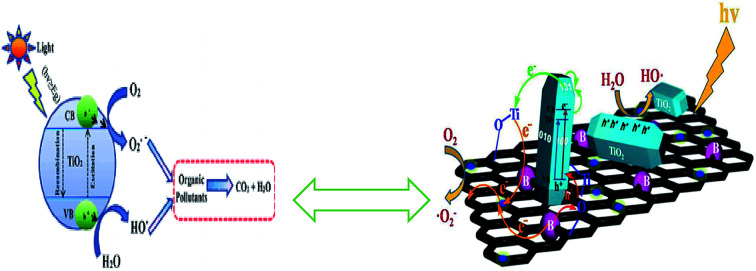

## 1. Introduction

Currently, research efforts have shifted toward semiconductor photocatalysts such as TiO_2_, ZnO, g-C_3_N_4_, *etc.*, due to the ability of these metals to convert pollutants into CO_2_ and H_2_O for environmental applications such as biofuel production and wastewater treatment. For modest and cost-effective treatment, additional efficient photocatalysts are highly preferred to parallel the extensively used TiO_2_. The field of water photocatalysis has experienced tremendous growth, following the discovery of photocatalytic H_2_ production in the 1980s and the photocatalysis and hydrophilicity of TiO_2_ films by Honda and Fujishima in the 1990s, and other scientists in the early 70s.^67–72^ Further studies have revealed that industrial applications in this area have been wonderfully accomplished since the early 90s based on the outcomes from these basic research efforts.^6,105–107^

Photocatalysis is the speeding up of oxidation and reduction reactions, brought about through the activation of a catalyst consisting of a semiconductor either alone or in combination with metal/organic/organometallic promoters, through light absorption and the subsequent charge and/or energy transfer, which can lead to the transformation of a pollutant. It must be noted that during the photocatalytic reaction, two actions must occur simultaneously in order for the successful production of reactive oxidizing species to occur. Typically, the first involves the oxidation of dissociatively adsorbed H_2_O by photogenerated holes, the second involves the reduction of an electron acceptor (typically dissolved oxygen) by photoexcited electrons; these reactions lead to the production of a hydroxyl and superoxide radical anion, respectively.^68,69,97,103,104^ In the area of photocatalysis, energy saving green tools are the ultimate, in accordance with photoinduced water cleavage to TiO_2_ electrodes, a phenomenon discovered by the pioneers in the field.^1^ It is believed that TiO_2_ photocatalysis is currently one of the best in the recent research in nanoscience and nanotechnology.

There have been many successful reports on the potential applications of TiO_2_ photocatalysts in water and environmental purification and management in the past few decades. In solving the recent issues related to energy and environmental purification, heterogeneous metal oxide semiconductor photocatalysts are best known. Due to the low-cost, nontoxicity, chemical stability and high efficiency of TiO_2_, it is unquestionably one of the most considered materials by researchers in the field of photocatalysis. However, the low quantum efficiency of TiO_2_ in photocatalytic mechanisms and the ineffective use of visible light for H_2_ harvesting, which mainly comes from its high recombination rate of photogenerated electron–hole pairs and wide band gap, have become problematic in prospective applications.^9,73,74^

Scientists in the field have adopted various approaches for the improvement of the photocatalytic efficacy of TiO_2_. These strategies can be summarized as morphological modifications, such as increased surface area and porosity, or as chemical modifications, by the incorporation of additional components into the TiO_2_ structure. Three basic strategies have been adopted for the structural modification, namely, doping with metallic/non-metallic elements or co-doping of metallic and non-metallic elements,^9,108–110^ modification through the introduction of defects such as oxygen vacancies and Ti^3+^ in the band gap,^111,112^ and surface modification by treatment techniques.^113–115^ Currently, the attention is centered on oxygen vacancies, which occur naturally in oxides, due to the vital role they play in the physical characteristics of materials; oxygen vacancies may be positive or negative. Oxygen vacancies occur when the number of oxygens in a particular compound is less than what it is supposed to be to make a perfect crystal lattice. This results in materials such as ZnFe_2_O_4_, where ZnO and Fe_2_O_3_ are chosen as starting compounds.

This review is centered on the ability and effectiveness of the prospective applications of oxygen vacancies and photocatalysis using TiO_2_ composites for energy, wastewater and environmental treatment in order to design future implementations to solve environmental problems. This is attractive for ecological uses such as water purification, purified biofuel production and wastewater treatment. Researchers in this field are doing their best in one way or the other to use these composites to solve the environmental crises. This discussion tackles the use of oxygen vacancies of TiO_2_ nano-based composites in photocatalysis to solve environmental concerns such as water treatment and related issues.

## 2. Review

### 2.1. Photocatalysis

**Fig. 1:**
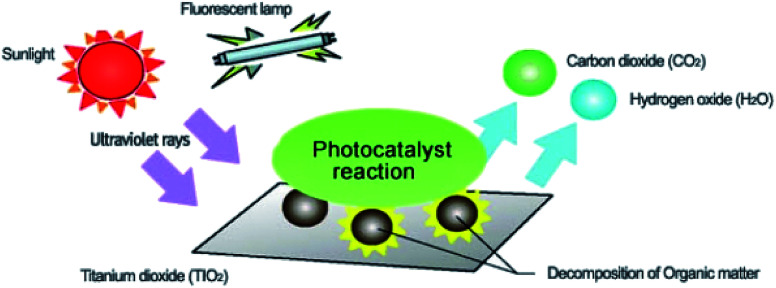
Schematic diagram demonstrating the photocatalysis mechanism.

Environmental remediation technology such as aromatic oxygen vacancy compounds and NO_*x*_ have been suggested by scientists in recent years for the treatment of urban pollution; thus leading to the use of photocatalytic self-cleaning and depolluting constituents. The photocatalytic properties of a thin layer of TiO_2_ entrenched in paints or concrete or placed at the surface of the particle influences the choice of these viable products. Photocatalysis is designed to harvest visible light (the major component of solar radiation that reaches the Earth's surface) using photocatalysts to drive chemical transformations (Fig. 1 and 3). The use of TiO_2_ photocatalysts as an evolving pollution control technology has been stated in numerous fields of science.^9,22,31,37,38^ It, therefore, seems that the actual influence and efficiency on the quality of water of these novel advancements have not been fully demonstrated to date but it has been revealed in a very restricted way. Photocatalysis embraces a class of reactions that use a catalyst activated by light and the decomposition of organic composites into water and carbon dioxide, leading to the fascinating properties of surfaces covered with a photocatalyst; these can shield against coating by fouling matter, are self-cleaning, antibacterial and viricidal.

**Fig. 2:**
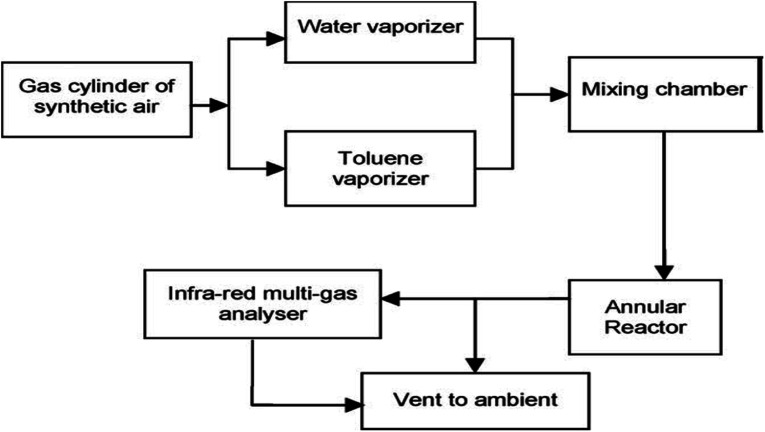
Flow diagram of the photocatalytic reaction system set-up.

**Fig. 3:**
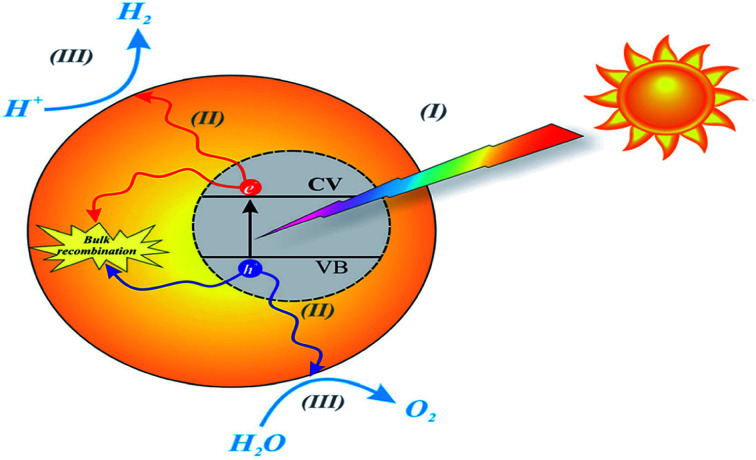
Schematic diagram of the basic mechanisms of the photocatalytic activity of water splitting. Reproduced with permission.^64^ Copyright 2015, *Nanoscale*. The Royal Society of Chemistry.

Heterogeneous photocatalysis in gas and liquid phase remediation has been intensively described by many researchers. Typically, the complete procedure is made up of five separate steps (Fig. 2): allocation of the reactants in the gas or liquid phase to the surface, adsorption of at least one of the reactants, reaction in the adsorbed phase, desorption of the product(s), and removal of the products from the interface region.

The third step is where the photocatalytic nature of certain metal oxides plays a role despite all the steps usually found in all heterogeneous processes. Semiconductor catalysts such as TiO_2_, ZnO, ZrO_2_, CeO_2_*etc.*, with photons carrying energy equal to or in excess of its band gap, create electron–hole pairs similar to photoinduced electron transfer and the absorption of light promotes one electron into the conduction band. The oxide may transfer its electron to any adsorbed electron acceptor (thereby promoting its reduction), while the hole (or the electron vacancy) may accept an electron from an adsorbed donor (promoting its oxidation).

**Fig. 4:**
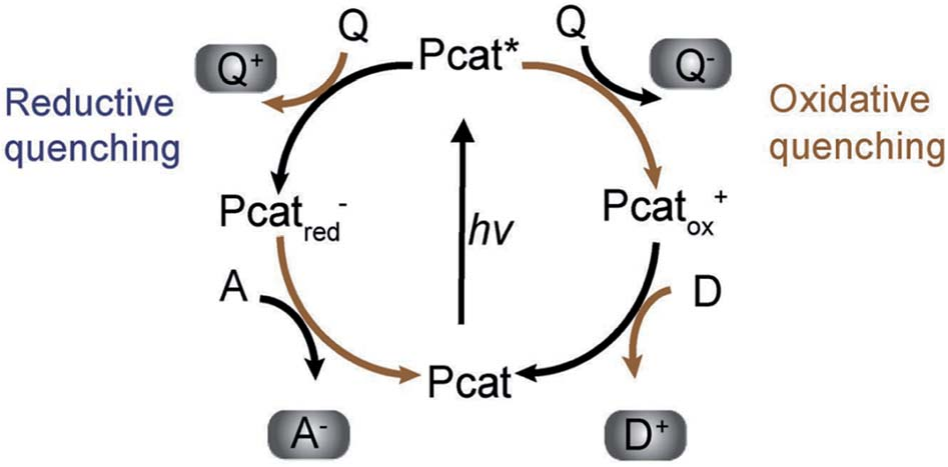
Photoredox catalysis by the photocatalyst. The oxidation steps are portrayed on the right; the reduction steps are shown on the left. Pcat: photocatalyst, Q: quencher, D: donor, A: acceptor. Reproduced with permission.^62^ Copyright 2009, Wiley-VCH Verlag GmbH & Co. KGaA, Weinheim.

TiO_2_ photocatalysis happens when the energy of the photons is enough to promote the electrons in the valence band to jump to the conduction band; this occurs in three steps:

(a) Photon absorption and electron–hole pair generation.

(b) Charge separation and migration to surface reaction sites or to recombination sites.

(c) Surface chemical reactions at active sites containing donor oxidation at valence-band holes and acceptor reduction at electron centers (Fig. 4).

**Fig. 5:**
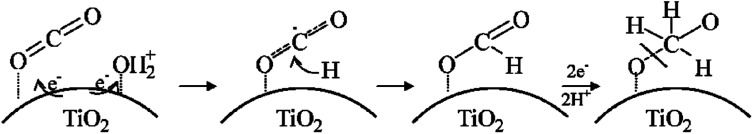
The mechanism of photoreduction of CO_2_ to the methoxyl radical on TiO_2_ in the presence of water during the photocatalytic reaction. Reproduced with permission.^63^ Copyright 2011, American Chemical Society.

Numerous defects associated with photocatalytic principles have been identified by researchers. During photocatalysis, cation radicals^17^ can be produced by injecting charge from excited molecules into the conduction band of TiO_2_ (Fig. 4 and 5).

Irradiation is usually the starting process in TiO_2_ photocatalysis; thus, the excitation of electrons by photons at the ground state is the prerequisite. Periodically, the excitation stage and thus the photoexcitation of electrons at the ground state also occurs in most of the materials adsorbed on the surface of the semiconductors;^97^*e.g.*, the reaction occurring in dye-sensitized solar cells.^42^ There are different pathways that are mainly experienced by the charge carriers. Many of the individual materials such as TiO_2_ are mostly used for water splitting, oxidation/reduction, (Fig. 5) in both suspension and electrode systems.

**Fig. 6:**
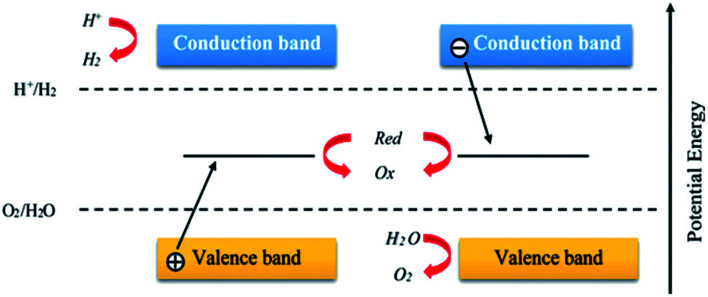
Schematic diagram showing the general water splitting ability of photocatalysts in the *Z*-scheme system. Reproduced with permission.^64^ Copyright 2015, *Nanoscale*. The Royal Society of Chemistry.

**Fig. 7:**
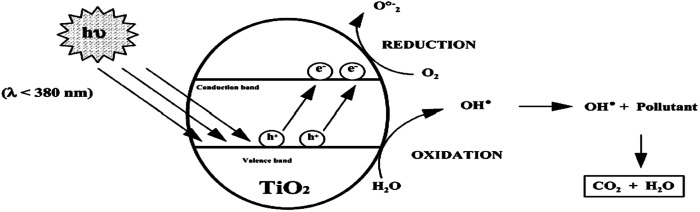
Schematic picture of the principles of the photocatalytic degradation of non-porous TiO_2_ particles.

Significant research attention is centered on metal oxides and silicates due to their photocatalytic activities and their wide range of applications in photocatalysis.^90^ Several semiconductors, such as TiO_2_,^91^ ZnO,^81^ Fe_2_O_3_,^82^ WO_3_,^75^ SrTiO_3_,^76^ NaTaO_3_,^77^ CdS,^78^ Ag_3_PO_4_,^75^ BiPO_4_,^79^ and g-C_3_N_4_,^80^ NiO,^83^ Cr_2_O_3_,^84^ Co_3_O_4_,^85^ Al_2_O_3_,^89^ are known photocatalysts, with their use for effective photocatalytic activities being dependent on their band gap^86–88^ (Fig. 6 and 7). The trapping experiments for holes and free radicals (ROS/RNS) are usually used to explain the photocatalytic schemes of photocatalysts such as TiO_2_. Also, the trapping experiments of holes, hydroxyl radicals (·OH), and superoxide radicals (·O^2−^) have been reported by many studies in photocatalysis as the main oxidative species that are found in photocatalytic processes.

**Fig. 8:**
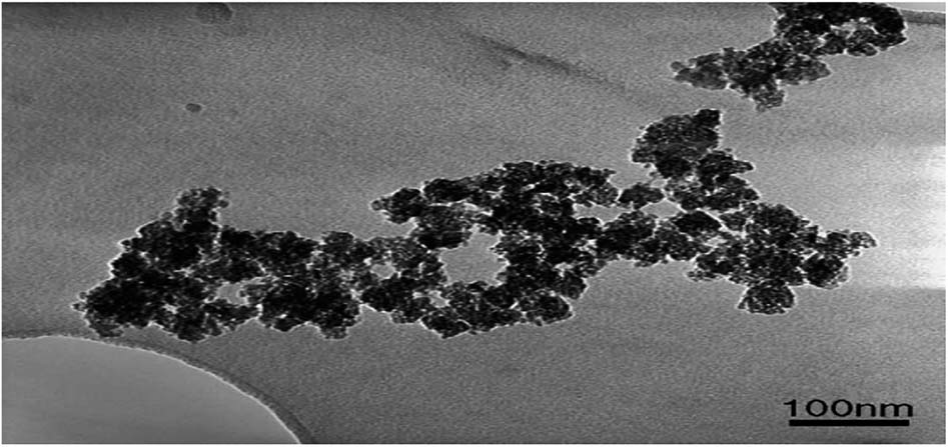
TEM image of the synthesized TiO_2_–SiO_2_ catalyst. Reproduced with permission.^66^ Copyright 2006, *Chemical Engineering and Processing*. Elsevier.

In instituting whether transformation or water splitting (Fig. 5) is really a photocatalytic activity, with direct activation of reactants and intermediates through visible light absorption, it is essential to establish that the photocatalyst absorbs the photons rather than the absorbents.^92,93^

Photoexcited charge carriers (Fig. 7) drive the conversion of water (Fig. 5) and carbon dioxide into H_2_, CO, CH_4_, and CH_3_OH related oxygenates and hydrocarbons^94–96^ during photocatalytic production.

#### 2.1.1. Hybrid non-porous TiO_2_ photocatalysts

The concentration of chemical compounds is determined by the rate of chemical reactions as described by Yu and coauthors.^55^ TiO_2_ has low adsorption ability, specifically for nonporous compounds, which is as a result of its polar structure. The synthesis of TiO_2_ with adsorbents is an important factor in improving the adsorption properties of TiO_2_ particles; the adsorbents hold the compounds on the adsorbent support. Yu and coauthors^45^ reported that an increase in the photoreaction rate comes about due to the formation of high environmental concentrations of the compounds around the nonporous TiO_2_ particle. Consequently, numerous schemes have been established to alter the properties of TiO_2_, anticipating the lengthening of the lifespan of the photogenerated electron–hole pairs and a narrower band gap. To extend the optical absorption to the visible region and the lifetime of TiO_2_, many successful studies by researchers in the field have centered on doping metal or non-metal elements into the TiO_2_ lattice to generate donors or acceptors in the band gap. However, there are substantial restrictions for this approach; the reduced incident photon to electron transformation efficiency in the UV light region and improved carrier recombination centers are the most challenging. Consequently, to heighten the useful application of TiO_2_, it is important to decrease the recombination and then increase the visible light absorption.^45^ Various adsorbents such as zeolum, alumina, silica, mordenite, ferrierite, and activated carbon, have been used as the support for TiO_2_ and they have shown that the hybrid photocatalysts are effective in achieving high decomposition rates of propionaldehyde in the air. Ao and Lee^46^ used activated carbon with TiO_2_ as the hybrid photocatalysts in their research and they made similar conclusions to those made by Yu and colleagues.^55^ The photodegradation efficacy of ZnO, when synergized with activated carbon as the adsorbent, was found to be higher compared to TiO_2_.

#### 2.1.2. Preparation of the TiO_2_ photocatalysts

Preparation methods (Table 1) for nano-based composites include the hydrothermal technique and water-in-oil microemulsion^29,49^, the sol–gel method^47^, and vapor decomposition of titanium alkyl oxides or TiCl_4_ in oxygen.^48^

**Table 1:** Comparison of selected preparative techniques for nonporous TiO_2_ photocatalysts

Preparation methods	Comparison
(1) Hydrothermal	(1) Preparation of TiO_2_ powder using this method usually uses liquid solutions as solvents to harvest the precursors. This usually occurs under increased temperature, <250 °C,^50^ and high-pressure conditions
	(2) Crystalline products are often formed (Fig. 8), considering the nucleation and crystal development. These products have different compositions, structures, and morphologies. TiO_2_ powders are obtained after critical washing and drying
	(3) NaOH or ethanol and water frequently serve as the solvents. TiCl_4_ and Ti(SO_4_)_2_ are also commonly used as precursors
	(4) The hydrothermal procedure is best used to improve crystallization on both the laboratory and commercial scales. The crystallization process^51^ is influenced by features such as reaction time, reaction temperature, the medium, and type of precursor
(2) Water-in-oil microemulsion techniques	(1) In recent years, scientists in this field have shifted a lot of attention to monodisperse nanoparticle preparation using this technique^49^ due to the following factors:
	(a) The water-in-oil microemulsion is thermodynamically stable^52^
	(b) It is also the optically isotropic dispersion of surfactant stabilized microdroplets of water in an external oil phase.^52^ These extremely dispersed nanosized microdroplets are well-matched for particle synthesis. This is due to the ability to control the microenvironment, the site for chemical reactions
3. Coating methods	(1) Studies have revealed that active commercial TiO_2_ powder possesses less photocatalytic activity than the TiO_2_ film.^53^ Photocatalytic activity is often influenced by coating the TiO_2_.^53^ TiO_2_ doped with elements such as C and N for doped TiO_2_ synthesis is less expensive^29^
	(2) There are two main coating methods:
	(a) Directly sintering or dip-coating (sometimes called wash-coating) the catalyst powders^54^
	(b) Formation of the TiO_2_ film on the support. This mostly uses the following preparative techniques:
	(i) Metal–organic CVD (MOCVD)^19^
	(ii) Chemical vapor deposition (CVD)
	(iii) Sol–gel^40,55^
	(iv) Spray coating

The TiO_2_ nanomaterial obtained is mostly based on the conditions used in its preparation. These include the following:

(1) Gas-phase method.

(2) Liquid-phase method.

#### 2.1.3. Point defects and standard specimens

The idea of defects primarily lies in the solid state physics explanation of lattice distortion. Lattice distortion is usually in the form of linear defects, point defects, three-dimensional valleys or hetero-impurities and two-dimensional flaws or interfaces. Fujishima and team found that point defects are well studied by scientists in the field and this delivers the principal understanding of the properties of lattice defects, consisting of magnetic, electrical, energetic, optical, and thermal characteristics in solids.^2^ OTDs are commonly accepted as point defects. In photocatalytic reactions, the point trap model is best used to analyze oxygen vacancies and OTD-related clusters.^43^ In the case of the synthesis process, lattice defects are certainly produced. These defects are thermal and preparation dependent.^43^ During material doping, intrinsic point defects are often observed in the lattice. These observed intrinsic point defects exist as atomic impurities, vacancies and interstitials. Studies have shown that these defects are scattered on flat surfaces as observed by STM images. The scattered distribution of these defects is noted to be the main reason for most of the increased photocatalytic activities.

In contrast, the presence of the coordinate and disordered defects in diverse samples makes it barely possible to exactly compare the photocatalytic properties. Under ambient conditions at room temperature, distinct particles are uncommon.^44^ To simplify the study on the molecular scale, much research has been devoted to the hypothesis pertaining to well-defined particles. Consequently, there is a strong call for the understanding of the nature of photocatalytic reactions regarding the preparation of comparable standard specimens.

### 2.2. Oxygen vacancy

**Fig. 9:**
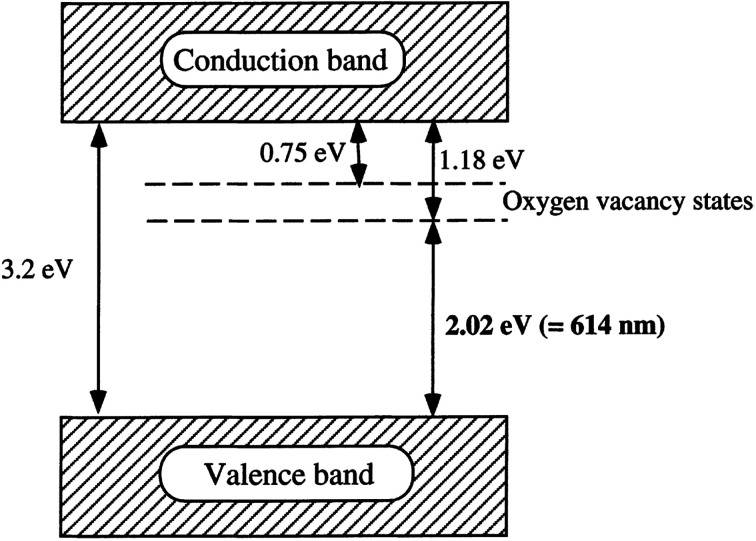
A proposed band structure model for the anatase TiO_2_ with oxygen vacancies. Reproduced with permission.^65^ Copyright 2000, *Journal of Molecular Catalysis A: Chemical*. Elsevier.

Oxygen vacancy occurs when the number of oxygens anticipated in a particular compound is less than what it is supposed to have to make it a perfect crystal lattice. Annealing in a reducing atmosphere is a prerequisite for the removal of oxygen from a compound made up of oxygen. Extra oxygenation requires annealing in an oxidizing atmosphere (O_2_). Simply put, this involves hole doping (the removal of two electrons as a result of adding one oxygen atom from the parent atom), and electron doping (addition of two electrons due to the removal of one oxygen atom from the parent atom). Magnetic and transport properties are mostly determined by the final material's structure, which is also dependent on its total charge/spin state. This idea is expected to be true in nanomaterials or bulk materials; however, anomalous properties are seen in nanomaterials, due to the ability of these materials to exist in new or active electronic states or environments (Fig. 9).^10^

Considering spinel type ferrites, the exchange of cations in the tetrahedral and octahedral sites in these magnetic oxides are well known to induce vacancies or lattice defects. There is, therefore, the inversion of normal spinel structures due to the occupation of most divalent transitional metal ions. At this point, the superexchange mechanism is produced due to the relative positions of both octahedral and tetrahedral sites of the atoms. Interatomic distances, anions, cations and bond angles are extensively close. The hopping mechanism that has a give-and-take of an e^−^ to a half-filled shell is often determined by the oxygen atom in the structure. Antiferromagnetic coupling to the unpaired spin of oxygen, as described by the Pauli exclusion and Hund's rule, or contribution to the net magnetism occurs due to an outstanding exchange with the closest neighbor transition metal. Negative and positive oxygen vacancies are two main types of oxygen vacancy. The concentration is centered on the oxygen vacancy, one of the natural spots in oxides, due to the vital role it plays in the physical characteristics of materials.

#### 2.2.1. Oxygen vacancies and the photocatalytic activity of nonporous, and doped TiO_2_

Recently, a novel technique involving positron annihilation lifetime spectroscopy was used to characterize oxygen vacancy associated with hydrogenation modified TiO_2_.^2^ This research revealed that hydrogenated TiO_2_ contains some larger vacancy clusters, a massive amount of small neutral Ti^3±^ oxygen vacancies and a small number of voids of vacancies. The emergence of new Raman vibrations occurred as a result of these defects, which led to the atomic lattice high-resolution transmission electron microscopy (HRTEM) images.^56^ The concentration of oxygen vacancies was measured by X-ray photoelectron spectroscopy (XPS) and this was found to be 3% in the TiO_2_ lattice. There was a remarkable increase in photocatalytic activity when the oxygen vacancies introduced by hydrogenation slowed the charge recombination; this was actually revealed by the photocurrent, photoluminescence (PL) spectroscopy, and degradation of methylene blue. Fujishima and research group adopted reduction in hydrogen atmosphere coupled with an impregnation process to prepare defective TiO_2_ with oxygen vacancies and Cu(ii) nanocluster modification.^2^ There was no improvement in the visible light from the TiO_2_ photocatalyst samples with optimum oxygen vacancy. Conversely, the photocatalytic enactments of TiO_2_ samples with a greater quantity of oxygen vacancies were sharply reduced; they even demonstrated much enriched visible light absorptions. Wang and group successfully used the hydrothermal method in their experiment to prepare platinum decorated TiO_2–*x*_N_*x*_*via* a facile one-pot route by using nanotubular titanic acid as a precursor.^3^ They further evaluated the photocatalytic performance of Pt/TiO_2−*x*_N_*x*_ for the visible-light-induced degradation of propylene in relation to the synergistic effect among single-electron-trapped oxygen vacancies and N plus Pt dopants. It was proposed that the synergistic effect among oxygen vacancies and dopants led to the enriched photocatalytic activity of the as-synthesized Pt/TiO_2−*x*_N_*x*_. The TiO_2_ photocatalyst spectral response to the visible light range is usually improved by impurity doping (Tables 2 and 3).^32,37^ In 2015, experiments performed by Park and group showed that the maximum entropy method (MEM) analysis and X-ray diffraction (XRD) used in the analysis of the changes in the *V*_O_ concentration as a function of the Co-doping level from ZnO Rietveld refinement generated the same results but X-ray photoelectron spectroscopy (XPS) confirmed a different outcome.^4^

**Table 2:** Comparison of the differences between doped TiO_2*x*_N_*x*_ and TiO_2_ photocatalysts (Fig. 10)

Doped TiO_2*x*_N_*x*_ photocatalyst	TiO_2_ photocatalyst
(a) Several nitrogen-doped TiO_2_ samples (Fig. 10)^7^ were found to photodegrade gaseous formaldehyde,^8^ acetaldehyde,^8,9^ acetone,^10^ 2-propanol,^11,12^ toluene,^8,13^ and methylene blue	(a) Pure TiO_2_ photocatalyst is usually less effective for photo depletion of methylene blue^39^
(b) TiO_2*x*_N_*x*_ (films and powders) has better photoactivity than TiO_2_ under visible light irradiation^29^ due to higher surface acidity (Fig. 10)^29,30^	(b) TiO_2_ has less photocatalytic activity under visible light irradiation
(c) The active wavelength of TiO_2*x*_N_*x*_, of less than 500 nm covers the main peak of the solar irradiation energy beyond the Earth's atmosphere (around 460 nm)^9^	(c) A similar active wavelength of 500 nm for TiO_2_ does not cover the main peak of the solar irradiation energy beyond Earth's atmosphere^9^
(d) Introduction of ZrO_2_ into TiO_2*x*_N_*x*_ exhibited higher porosity, higher specific surface area, and an enhanced thermal stability^14^	(d) This feature was absent in TiO_2_
(e) Decreases the deactivation of the photocatalysts^28^	(e) Deactivation of the surface occurs very quickly

**Table 3:** Typical metals for TiO_2_ photocatalyst doping

Metals	Properties	References
(1) Transition metal ions such as V, Cr, Mn, Fe, Co, Ni, or Cu	(1) Extend light absorption into the visible region	27 and 41
	(2) There is a considerable reduction in the photocatalytic activity in the UV region	
(2) Presence of metals, such as Li^+^, Zn^2+^, Cd^2+^, Ce^3+^, Co^3+^, Cr^3+^, Fe^3+^, Al^3+^, Mn^2+^ and Pt	(1) Considerably change the photocatalytic activity of TiO_2_	39 and 41
	(2) Sol–gel systems can be used to prepare the Mn^+^/TiO_2_ layers for phenol degradation	
(3) The presence of Co^3+^, Cr^3+^, Ce^3+^, Mn^2+^, Al^3+^ and Fe^3+^ ion (5 mol% Mn^+^: Ti^4+^)	(1) Has an opposing influence on the photocatalytic activity of the TiO_2_ photocatalyst	41
	(2) There is a decline in the photocatalytic activity of TiO_2_ under UV irradiation	
	(3) These metal ions act as recombination sites for the photogenerated charge carriers	

The Wendt group's experiment^5^ on adsorption and desorption, where they used oxygen and water as probe molecules that were observed in STM images to bridge oxygen vacancies, and oxygen atoms on surface Ti atoms, reported the production of pure and reduced TiO_2_ surfaces. Their work further addressed the criteria to determine the cleanliness by STM imaging.^5^ Yang and colleagues in their research in 2010 on Chinese housing energy and environment introduced a novel technique for the characterization of oxygen vacancy associates in mostly hydrogen modified TiO_2_ photocatalyst using positron annihilation lifetime spectroscopy (PALS). They concluded that small neutral Ti^3±^ oxygen vacancies (large quantity), vacancy clusters (appreciably large in size), and voids of vacancies (just a few of them) were actually in hydrogenated TiO_2_. In the research by Fujishima and coworkers,^2^ there was a remarkable improvement in the photocatalytic activity of 2% WO_3_–TiO_2_ catalysts when an appropriate oxygen vacancy was employed by using Fe^3+^ as an electron acceptor under UV irradiation in 12 hours.

Researchers have found out that the controlled combustion of Ti metal in a natural gas flame can also be used to synthesize chemically modified n-type TiO_2_ by using carbon as a doping agent.^8,15^ Inhibition of the charge recombination and trapping of the photoexcited electrons using these doped impurities (Table 3) also helps to increase the photocatalytic activity.^33–36^ There are three methods for preparing the visible light responsive photocatalyst, namely, doping TiO_2_ with transition metal ions, doping nitrogen into TiO_2_ and utilizing sensitizing dyes.

Semiconductors such as sensitizing dye (higher band-gap), change the electron-transfer processes during the photocatalytic reaction as described by Vinodgopal and Kamat in the principle of photosensitization of a semiconductor.^16^ Dye cation radicals^17^ can be produced by injecting charge from the excited dye molecule into the conduction band of TiO_2_.

#### 2.2.2. Quantitative analyses of oxygen vacancies

The quantitative analysis of oxygen vacancies has been successfully researched by many scientists in the field. Researchers have adopted the following oxygen vacancy quantitative analysis tools:

(1) The maximum entropy method (MEM).

(2) X-ray diffraction (XRD).

Most research efforts have also proposed a method for the analysis of oxygen vacancies using conventional XRD and MEM techniques (Table 4).

**Table 4:** Comparison of selected quantitative analysis tools for oxygen vacancy

Analysis tool	Comparison
(1) X-ray diffraction (XRD)	(1) X-ray diffraction (XRD) is a simple and useful tool for the analysis of oxygen vacancy because it reveals the crystal structure and the electron density distribution of periodic arrays of atoms
	(2) Analysis of X-ray diffraction data using Rietveld refinement has been attempted for the quantitative analysis of oxygen vacancies in terms of oxygen site occupancy
	(3) X-ray diffraction requires the use of neutrons or synchrotron X-rays
(2) Maximum entropy technique	(1) The maximum entropy method (MEM) is also a suitable tool for the analysis of oxygen because it uses the more precise Rietveld refinement that resolves summation-terminated errors and affords a better structural model
	(2) The maximum entropy technique presents insignificant modeling errors *via* the least-biased electronic reconstruction of X-ray diffraction patterns in real space

The efficiency of a particular photocatalyst depends strongly on the competence of electron–hole pair separation and the adsorption ability of gaseous oxygen vacancy compounds. Coupling TiO_2_ catalysts^18-20^ with other semiconductor oxides or depositing metals or doping TiO_2_ photocatalysts with some other metal ions help to improve its photocatalytic activities. Studies have^18^ established that the photoreactivity for both oxidation and reduction significantly improved when they doped with Fe^3+^, Mo^5+^, Ru^3+^, Os^3+^, Re^5+^, V^4+^, and Rh^3+^ at 0.1–0.5%, but Co^3+^ and Al^3+^ doping decreased the photoreactivity.^22^ When the active sites on the reaction surface^21^ of a gas–solid photocatalyst is reduced, the activity also decreases.^21^ It can be concluded that the activity of catalysts depends on their lifetime, which is potentially essential to the economic process.^21^ Studies on toluene,^23,24^ ethanol^25^ and trichloroethylene, dimethyl sulfide, and trichloropropene^22^ have found out that deactivation in these compounds is really uncommon. Sauer and Ollis^26^ in their work stated that particle materials block pores on the photocatalysts' surfaces, hence, change the surfaces of these catalysts.^27^ The chemical or physical adsorption of organic substrates on the TiO_2_ matrix is improved when of lanthanide ions, for instance, La^3+^, Eu^3+^, Pr^3+^, Nd^3+^, and Sm^3+^ amalgamated into the matrix^19^.

#### 2.2.3. The function of the TiO_2_ oxygen vacancy in the energy structure

The pristine energy structure of most outstanding nanomaterials, such as TiO_2_ is fabricated by the conduction band and valence band. It is noted that the development of the main active sites, mostly for visible light absorption and the band gap is supported by these energy levels. Lv and group stated that the function of oxygen vacancies as the visible light response sites is due to the defective TiO_2_ surface during visible light irradiation.^57^ The influence of the energy structure of the TiO_2_ photocatalyst on the charge carrier migration and light absorption is very high (this happens when there is the removal of oxygen atoms; Ti^3+^ is not involved and when there is the occurrence of oxygen vacancies). This calls for urgent, precise calculations of the defect state in TiO_2_ energy structure.^2^ Fujishima and group have reported that the defect states vary remarkably in the band gap. Recent work^58^ reported lower energy (for all Fermi-level positions in the band gap) in oxygen vacancies. It has been proven that within the band gap of TiO_2_, the oxygen vacancy usually forms a mid-gap electronic state and, therefore, TiO_2_ functions as the donor. This novel trend was supported by research performed by Eun Chang and colleagues.^39^ These scientists further summarized energy levels of oxygen vacancies in TiO_2_. The paradox of the intensely localized small polarons and the delocalized free polarons failed to be explained vividly by isolating electronic bands in many experiments and this led to hybrid function, which has been a remarkable theory. In the electronic band, it was reported that there exist two types of hybrid functions:

(a) Between the electrons and the conduction band in the presence of delocalized free electrons.

(b) Between the electrons and the oxygen vacancies in the form of oxygen vacancy complexes and the ionized shallow-donor impurities.

Considering the theoretical viewpoint, the highest occupied molecular orbital and the lowest unoccupied molecular orbital often shifts due to the influence exerted by the defect states.^59^ It was realized from experiment^59^ that an occupied defect state of 0.7 eV below the bottom of the conduction band was decreased by the oxygen vacancy.^7^ Research on the characterization analysis of anatase, rutile, and brookite showed that anatase and brookite were made up of oxygen vacancies, which remarkably improved the photocatalytic activity. In conclusion, it is presumed that the difference in the absorptions on the surfaces of the active sites and the reactants (CO_2_, H_2_O, CO_2_^−^, and CO) were due to oxygen vacancies.

#### 2.2.4. The influence of oxygen vacancies on charge transfer

**Fig. 10:**
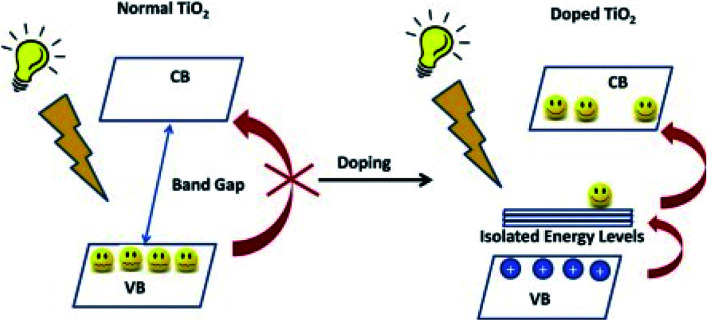
Schematic diagram showing the activities of doped nonporous TiO_2_ and nonporous TiO_2_.

**Fig. 11:**
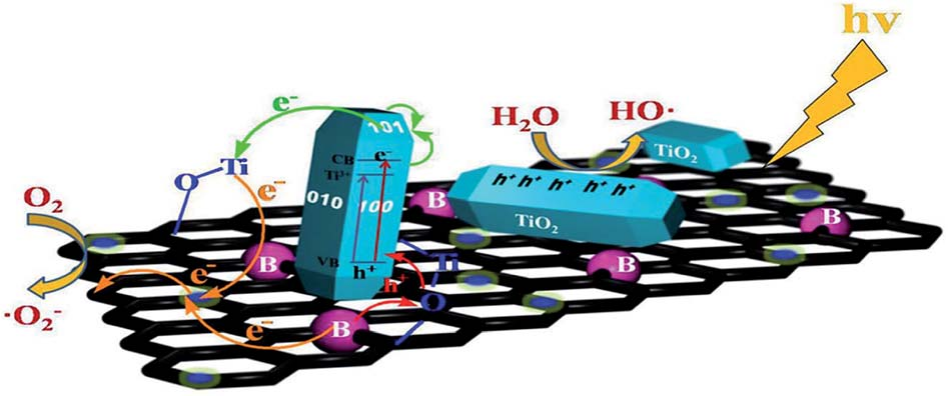
Diagram showing the charge transfer of the energy structure of TiO_2_.

In situ-EPR is a versatile technique for monitoring the separation and transfer of photogenerated electrons in semiconductors based on oxides^101,102^ or carbon nitrides^98^ since electrons excited to the conduction band can be trapped at oxygen vacancies^100^ and carbon defects.^99^ The promotion from the highest occupied molecule orbitals to the lowest unoccupied molecule orbital with light irradiation causes the rapid movement of an excited single electron in response to the applied electric field; *i.e.*, the voltage supplied by a power source or difference in potential between the energy structure of TiO_2_ and the redox potential of the adsorbed species (Fig. 10). Due to small lattice distortions in electron creation, the Franck–Condon factor is mostly small.^60^ According to the Marcus–Hush electron transfer theory, the transfer of charge carriers is restricted by the disappearing reorganization of energy but it usually follows a band model and hopping model (Fig. 11).

Lattice distortion makes use of the original energy provided by an election or a photon. The swiftly vanishing approach for the charge carriers^7^ has been reported by many scientists as annihilation at the recombination center. Mitsuhara and coworkers' studies^43^ on semiconductors led to a proposal of three recombination mechanisms (Table 5).

**Table 5:** Three recombination mechanisms, their properties and limitations

Recombination mechanism	Properties	Limitations	References
(1) Band-to-band recombination	(1) It occurs between the excited electron and the hole lying in the empty valence band	(1) The production of available electrons and holes limits this reaction	61
	(2) It is second order to the concentration of the charge Carrier		
(2) Trap-assisted recombination	(1) This mechanism transpires with the help of the “trap” state	(1) Shockley–Read–Hall model (SRH model) confirmed that the concentration of charge carriers hinders this reaction or mechanism	
	(2) It happens between the excited electrons and holes in the valence band		
(3) Auger recombination	(1) This usually happens during the recombination process of the excited electron and hole		20
	(2) Releasing the energy to improve the energy of another electron or hole		

#### 2.2.5. Applications of oxygen vacancies in photocatalysis

The impact of oxygen vacancy in photocatalysis includes the following:

(i) To alter the band energy structure of the pristine TiO_2_ as the defect states.

(ii) To trap charge carriers in the migration pathways as the electron pool or recombination center.

(iii) To power the adsorption of reactants (*e.g.*, H_2_O, O_2_, CO_2_, and organic pollutants) as the active sites.^61^

## 3. Conclusion

This systematic review sums up the novel developments in the photocatalytic applications of TiO_2_-based composite photocatalysts with oxygen vacancies in the areas of energy, wastewater treatment and environmental purification. Nonporous TiO_2_ has revealed its greatness as one of the best candidates in designing and engineering advanced composite photocatalysts. There is little doubt that the considerable progress in TiO_2_ nano-based composites will continue in the near future. More studies are necessary in order to make full use of the excellent properties resulting from the oxygen vacancy of the nonporous TiO_2_ photocatalysts.

## Conflicts of interest

The authors declare that they have no conflict of interest.

## Supplementary Material
